# Pause

**DOI:** 10.15694/mep.2019.000176.1

**Published:** 2019-09-17

**Authors:** Roger Khouri

**Affiliations:** 1 University of Texas Southwestern

**Keywords:** Residency, Communication, Clinical Training, Empathy, Burnout

## Abstract

This article was migrated. The article was marked as recommended.

In this perspective piece, a resident physician discusses the pressure to provide care as quickly as possible and how this pressure conflicts with the goal of providing humanistic care. The lack of time to connect and empathize with patients compromises care and leads to physician burnout. Taking a moment to pause, connect, and empathize may be the most time efficient aspect of any encounter.

## Pause

As a teenager, I chose to pursue medicine over engineering because of the human connection that comes from caring for the sick. Despite my love for math and science, I did not want to spend my time in front of a computer, collecting and processing data. I wanted to support people through their toughest times, bring them hope, and rejoice with them in their recoveries.

In medical school, we learned not to focus so narrowly on treating the disease that we forget to treat the patient. Patients were not data registries that needed to be processed and solved; they were people who needed to be talked to and cared for. I took pride in connecting with people at a human level and upholding the intrinsic value of their experiences. I looked forward to bringing these ideals to life with my own patients.

As a junior urology resident, I quickly learned that, between consults, surgeries, and inpatient care, a typical day left no time for these types of connections. Between OR cases, I put in notes and orders and consented the next patient. Any additional consults or inpatient care needed to be dealt with in the remaining few minutes before the next OR case. When preparing to see a consult, I first mined through the EMR to understand the history and reason for consult and prepare my note. This left a tiny window of time for the patient interview and physical exam. In an effort to avoid being late for the next OR case, I learned to think of the interview and physical as another task to collect data as quickly as possible. Connecting with patients on a personal level takes much time and yields little data.

When the healthcare system pushes physicians to be more and more efficient, the easiest corner to cut is the patient connection because it can be time consuming, and its benefits are not immediately obvious. However, a growing body of evidence suggests that patients whose physicians take the time to empathize with them have improved outcomes ( Neumann *et al*., 2007; Hojat *et al*., 2011; Rakel *et al*., 2011; Del Canale *et al*., 2012). Moreover, the connections physicians make with patients provide meaning to the long and stressful days, and thereby, allow us maintain resilience in an era of physician burnout.

Unfortunately, academic medical centers are not immune to these pressures for efficiency and productivity. Unlike medical students, surgical residents are taught to work as quickly as possible. We learn that long talks with patients can lead to concerns about our efficiency. Programmed to succeed, we practice medicine like data analysts, collecting and processing data, one patient at a time. This dehumanization, coupled with the long and stressful days, leads to burnout.

In every patient encounter, physicians decide to either take the extra time and connect with the patient or simply collect and process the data and push to stay on schedule. For better or worse, I learned to pause. For a few moments at the end of every encounter, I consciously take my foot off the gas pedal. I turn the data-collecting machine off and connect with the patient on a human level. I tell them that I understand that what they are going through is difficult and that I will support them through it. They then typically tell me what worries them most about their condition and that they trust me to do what is right for them. These moments make the long and stressful days worthwhile and motivate me to become a better physician.

Medicine is not engineering. There is much more to caring for a patient than simply collecting and processing data. Empathy makes medicine human. Omitting it compromises care and leads to physician burnout. Taking a moment to pause, connect, and empathize may be the most time efficient aspect of any encounter. In this era of burnout, we must teach the next generation of physicians that we don’t have time not to pause.

## Take Home Messages

• Residents are incentivized to maximize efficiency and spend as little time as possible communicating with patients
• This emphasis on efficiency over empathy leads to poor patient outcomes and physician burnout
• Training this way will produce a generation of physicians ill-equipped to address the human needs of the population

## Notes On Contributors

Dr Khouri was born in St. Louis and raised in Miami. He went to college at Wake Forest University and medical school at the University of Michigan. He is a PGY-3 urology resident at the University of Texas Southwestern. After residency, he plans to complete a fellowship in reconstructive urology and work as an academic urologist.
