# Supplementary material for: Pause
Source: MedEdPublish (2016). 2019 Sep 17;8:176. [Version 1] doi: 10.15694/mep.2019.000176.1 (PMC10712516; doi:10.15694/mep.2019.000176.1)
Supplement: Peer review history

# Reviewer report: Reviewer response for version 1

**DOI:** 10.21956/mep.19881.r30069

**Published:** 2020-09-03

**Authors:** Thomas Puthiaparampil

**Affiliations:** 1 UNIVERSITI MALAYSIA SARAWAK

This review has been migrated. The reviewer awarded 5 stars out of 5 Congratulations, Dr. Roger Khouri. Your article really made me pause. The title itself is irresistible. I too feel doctor burnout is a real issue in modern times as performance and time management are given more importance than patient satisfaction. While assessing medical students also we do not give much marks for the aspect of empathy, but I realize it is really important to give more value to this aspect. As one of the reviewers mentioned, I too plan to share this article with our medical students. The article is very well written, to the point, and gave me many statements which are worth quoting. I hope the aspect of empathy will be given the weight it deserves in the assessments of medical graduates and it is given adequate importance in their training before that. After all, doctors seldom cure patients, but they always have the possibility and potential to give hope and consolation to their clients.

Reviewer Expertise:

NA

No decision status is available

# Reviewer report: Reviewer response for version 1

**DOI:** 10.21956/mep.19881.r30068

**Published:** 2020-06-11

**Authors:** Wafaa Mowlabaccus

**Affiliations:** 1 University of Mauritius

This review has been migrated. The reviewer awarded 4 stars out of 5 As a medical student, I find this article thought provoking and insightful. While in medical schools, much emphasis is attributed to a good doctor-patient relationship, this article has eloquently portrayed the reality versus what we are usually taught.

Reviewer Expertise:

NA

No decision status is available

# Reviewer report: Reviewer response for version 1

**DOI:** 10.21956/mep.19881.r30071

**Published:** 2019-12-10

**Authors:** Simonetta Ausoni

**Affiliations:** 1 Department of Biomedical Sciences- University of Padova- School of Medicine

This review has been migrated. The reviewer awarded 5 stars out of 5 I read this document with great interest, I will download it and give it to my medical students as a point of reflection. As a basic scientist and a teacher in the medical school, I’d like to contribute by looking at the point from a different perspective. When the academic environment pushes students to be obsessively on track with their exams, when not being on track may compromise career, when teacher/student formative relationship is restricted to a tiny space, it is obvious that there is a price to pay. Students are exhausted by this enormous pressure. Sometimes they become doctors without even really knowing what they want to become, because they haven't had enough time to think about it. These students will be very good working under pressure because they have been educated with the same style. And if it is true that doctors run the risk of burnout, I can say that even students run the same risk and sometimes they do not even exceed it.

Reviewer Expertise:

NA

No decision status is available

# Reviewer report: Reviewer response for version 1

**DOI:** 10.21956/mep.19881.r30067

**Published:** 2019-10-31

**Authors:** Alexander Woywodt

**Affiliations:** 1 Lancashire Teaching Hospitals NHS Foundation Trust

This review has been migrated. The reviewer awarded 5 stars out of 5 I really enjoyed reading this personal view piece. I agree with the other reviewers that the author's statement that "medicine is not engineering" sums it up very well. I am curious as to how the author makes this work in the real world. How long are the patient slots are on the clinic template and how much time can the author spend on taking the foot off the gas pedal? Also it would be nice to see a more detailed reflection on how care providers can connect. Perhaps there are other ways of doing this other than empathy? I often connect with patient through the artwork in my consulting rooms or when I take an occupational history. Finally the author focuses on time pressures in a busy clinical environment but isn't lack of continuity one major obstacle for health care providers to connect with their patients? Perhaps its stating the obvious but the author could briefly reflect on lack of continuity and the increasing factory-like character of medicine as a factor in this.

Reviewer Expertise:

NA

No decision status is available

# Reviewer report: Reviewer response for version 1

**DOI:** 10.21956/mep.19881.r30070

**Published:** 2019-09-22

**Authors:** Helena Filipe

**Affiliations:** 1 Hospital of the Armed Forces/PL-EMGFA

This review has been migrated. The reviewer awarded 5 stars out of 5 The statement “ Medicine is not engineering” summarizes the core of this meaningful, very well written article. In the category of “perspective”, the article reads well, with a fluid writing and articulated ideas portraying the Author’s view, thoroughly supported on evidence.The chosen topic is critical within the healthcare delivery context and has significant impact on medical education in terms of available time and motivation of the clinical educator to impart experience as well as the kind of role modeling thus possible to offer. The Author emphasizes the relevance of the chair time in the clinical encounter to enable the doctor-patient relationship development. I completely agree there should be no subtracting or delegating this fundamental component of medical care in order to reach the best patient outcomes and ultimately the community health well being. This is a physician’s role and responsibility.The Author underlines well how it serves both parties maximizing the physician’s job satisfaction and reducing burnout liabilities as also nurtures patients’ compliance for clinical best outcomes. “Medicine is not engineering”. Medicine is about people and breathes through meaningful professional relationships. Well done!

Reviewer Expertise:

NA

No decision status is available

# Reviewer report: Reviewer response for version 1

**DOI:** 10.21956/mep.19881.r30072

**Published:** 2019-09-21

**Authors:** sathyanarayanan varadarajan

**Affiliations:** 1 SRM Medical College, SRM University

This review has been migrated. The reviewer awarded 5 stars out of 5 In this important article of personal view, the author raises a significant issue on spending time for patient connection and empathy. He feels that we as doctors are programmed to succeed, so we practice medicine like data analysts, collecting and processing data, one patient at a time. This dehumanization, coupled with the long and stressful days, leads to burnout.So the author learnt to pause for few moments, connected with the patients on the human level and communicated to them with empathy. He realized that these moments made the long and stressful days of him worthwhile and motivated him to become a better physician.He concludes strongly that empathy makes medicine human and omitting it compromises care and leads to physician burnout.He urges that we must teach the next generation of physicians that we don’t have time not to pause.I totally agree with him that Empathy and Connecting with patients are vital components of effective patient care and it’s high time now to take active steps to educate the coming generation of doctors to practice these.

Reviewer Expertise:

NA

No decision status is available
